# Hyperlipidemia and Platelet Parameters: Two Sides of the Same Coin

**DOI:** 10.7759/cureus.25884

**Published:** 2022-06-12

**Authors:** Anurag Singh, Anupam singh, Rashmi Kushwaha, Geeta Yadav, Tanya Tripathi, S C Chaudhary, Shailendra P Verma, Uma S Singh

**Affiliations:** 1 Pathology, King George's Medical University, Lucknow, IND; 2 Heamatopathology, King George's Medical University, Lucknow, IND; 3 Medicine, King George's Medical University, Lucknow, IND; 4 Hematology and Oncology/Clinical Hematology, King George's Medical University, Lucknow, IND

**Keywords:** platelet aggregation, platelet function, platelet volume indices, platelet, hyperlipidemia

## Abstract

Introduction: Hyperlipidemia is a disorder in which lipid and cholesterol levels in the blood are elevated. Diabetes, coronary heart disease, obesity, and hypertension are commonly linked to hyperlipidemia. Despite this, hyperlipidemia is a widely neglected illness, owing to its asymptomatic nature, ignorance of aberrant lipid profiles on screening, and economic issues in poor countries such as India. Platelets have been shown to have a role in the thrombus consequences of atheromatous damage in hyperlipidemic individuals by initiating and propagating atherosclerotic plaques. Platelets with bigger diameters are thought to be more metabolically, enzymatically, and functionally agile than platelets with lower sizes. In steady-state operation, these bigger platelets release more thromboxane B2 than regular platelets. Platelets with bigger sizes are more hemostatically active and hence have a higher chance of forming a thrombus and thromboembolism. The aim of this study was to compare the values of key platelet parameters and platelet function in hyperlipidemic patients with normal age and sex-matched controls.

Material and methods: A total of 100 individuals were included in this study, with 68 cases of hyperlipidemia and 32 controls having normal lipid profiles. Platelet volume indices (PVI) such as platelet count (PC), mean platelet volume (MPV), platelet distribution width (PDW), platelet large cell ratio (P-LCR), plateletcrit (PCT), and platelet function (platelet aggregation with adenosine diphosphate, ADP) were compared between hyperlipidemia patients and age sex-matched controls with normal lipid profiles.

Results: The cases had a statistically significant higher mean MPV (10.55 ± 1.81), PDW (14.93 ± 2.82), and P-LCR (30.97 ± 11.74) compared to mean MPV (9.35 ± 1.85), PDW (13.10 ± 2.60), and P-LCR (25.13 ± 12.23) of controls (p-value < 0.05). No significant difference was observed between the study group and control group with respect to mean PC and PCT (p-value > 0.05). In this study, there was a statistically significant increase noted in platelet aggregation percentage in hyperlipidemic patients than in the control group (42.03 ± 25.28 vs 31.25 ± 15.11) (p-value < 0.05).

Conclusion: To conclude, platelet parameters are a significant, easy, and cost-effective method for predicting future acute episodes in hyperlipidemic patients that should be utilized more widely. To avoid vascular events, these individuals may require higher antiplatelet dosages and more rigorous hyperlipidemia therapy.

## Introduction

Hyperlipidemia is a disorder in which lipid and cholesterol levels in the blood are elevated. It is also known as dyslipidemia, which refers to a variety of lipoprotein metabolism abnormalities. Although an elevated amount of low-density lipoprotein (LDL) and cholesterol in the blood is regarded to be a conventional risk factor for atherosclerosis [1]. Hyperlipidemia is defined as high total cholesterol (TC) and/or triglycerides (TGs) and/or low levels of high-density lipoprotein (HDL) and cholesterol. Hyperlipidemia is described by the American Heart Association as an elevated amount of fats in the blood. Cholesterol and TGs are examples of these fats. Hyperlipidemia appears to be a significant risk factor for coronary heart disease, stroke, and heart attack [2]. Diabetes, coronary heart disease, obesity, and hypertension are commonly linked to hyperlipidemia [3-4]. Despite this, hyperlipidemia is a widely neglected illness, owing to its asymptomatic nature, ignorance of aberrant lipid profiles on screening, and economic issues in poor countries such as India. Platelets have been shown to have a role in the thrombus consequences of atheromatous damage in hyperlipidemic individuals by initiating and propagating atherosclerotic plaques, although their primary purpose is to maintain hemostasis by initiating blood coagulation [5]. Platelets bind to regions of vascular endothelial injury and release mitogenic factors including platelets derived growth factor (PDGF) and tumor growth factor (TGF), which contribute to the early stages of atherosclerosis [6]. Platelets with bigger diameters are thought to be more metabolically, enzymatically, and functionally agile than platelets with lower sizes [7]. In steady-state operation, these bigger platelets release more thromboxane B2 than regular platelets. Platelets with bigger sizes are more hemostatically active and hence have a higher chance of forming a thrombus. Platelet size or mean platelet volume (MPV) is, therefore, used to indirectly quantify platelet activity [8]. Platelet count (PC) and platelet volume indices (PVI), such as MPV, platelet distribution width (PDW), and platelet-large cell ratio (P-LCR), are now regularly accessible in most clinical laboratories at no additional cost. The simple availability of direct measures of PVI has motivated research on platelet activation's role in the etiology of hyperlipidemia-related illnesses. Increased MPV in hyperlipidemic patients has been linked to a variety of disorders, including coronary artery disease, myocardial infarction, and cerebral infarction according to previous studies [8-10]. Prostaglandin I2 (PGI2) and nitric oxide (NO) are inhibitors of platelet activity produced by endothelial cells of blood vessel walls in normal circumstances. PGI2 and NO work together to catabolize adenosine diphosphate (ADP) (platelet activator) to adenosine and have ecto-ADPase activity (platelet inhibitor). All three systems operate in concert to keep platelets from activating. When the endothelium of blood arteries is injured, these processes are disrupted, and the subendothelial matrix is exposed, allowing for powerful platelet activation and changes in platelet shape, resulting in the creation of a platelet plug to stop bleeding. The formation of a thrombus starts with the generation of a platelet plug, which is subsequently stabilized by the deposition of fibrin and leukocytes in the plug [11]. This study deals with testing PVI and platelet function in hyperlipidemia patients. The aim of this study was to compare the values of key platelet parameters and platelet function in hyperlipidemic patients with age and sex-matched controls.

## Materials and methods

Study setting

This was a prospective study conducted at the Department of Pathology in collaboration with the Department of Internal Medicine, King George’s Medical University Lucknow, a tertiary care hospital in North India. The study period was of one-year between January 2015 and December 2016. This study was approved by the ethics committee of King George’s Medical University, Lucknow on 04 December 2015 (No.7935/Ethics/15).

Inclusion and exclusion criteria

According to the third report of the National Cholesterol Education Programme (NCEP) evidence-based guidelines for cholesterol testing and management, the cases were chosen after being diagnosed as hyperlipidemic based on the laboratory reports of either hypercholesterolemia and/or high LDL levels and/or hypertriglyceridemia. Controls were those who were suspected of being hyperlipidemic but had a lipid profile within the normal range. The study excluded patients who had any cardiovascular illness, bleeding problems, or was using anti-platelets, anti-coagulants, or lipid-lowering medicines. Thrombocytopenia, anemia, cancer, pregnancy, recent blood transfusion history, infections, chemotherapy, and patients under the age of 20 years were also eliminated from the study group.

Study design

The study was a prospective observational study.

Data collection

For the collection of data, case sheets of the study population were used to record a detailed clinical history and laboratory findings. In this study, the impact of several platelet parameters (PC, MPV, PDW, P-LCR, and PCT) and function (platelet aggregometry with ADP) was studied in hyperlipidemic patients and age-sex matched controls. A total of 100 individuals were included in this study, with 68 acting as cases and 32 as controls. All individuals provided a complete clinical as well as current treatment history. The serum lipid profile of the patients and controls in this study was done, which included serum cholesterol, TGs, low-density lipid, high-density lipid, and very-low-density lipid. A complete blood count (CBC) was performed, including platelet parameters such as PC, MPV, PDW, P-LCR, and PCT. All participants had their platelets’ function tested using platelet aggregation with ADP. The sample was obtained from patients after they had fasted for 10-12 h, ensuring that the lipid profile values were unaffected. Some 8 mL of blood was extracted from the antecubital vein under aseptic condition after informed consent. Some 2 mL of blood was dispensed into a K3EDTA-containing vacutainer tube. Some 2 mL of blood was dispensed into a vial of plain serum. The rest of the blood was distributed into a vacutainer tube containing sodium citrate in a 9:1 ratio. Blood samples were tested within 2 h after receiving the samples. The blood lipid profile [TC, TG, LDL, and very low density lipoprotein (VLDL)] was analyzed using a semiautomated chemical analyzer (ELITech Group Clinical Systems- Selectra Pro-XL)AQ from serum centrifuged at 2500 g for 15 min. From blood in vacutainer tubes containing K3EDTA, a complete hemogram was performed on a five-part cell counter. Hemoglobin (g/dL), hematocrit, white blood cell (WBC), PC, MPV, PDW, P-LCR, and PCT were all measured. A light transmission aggregometer was used to study platelet aggregation in both patients and control platelet-rich plasma (PRP). PRP was produced by centrifuging venous blood in a trisodium citrate vial (9:1) for 5 min at 1000 rpm at room temperature (20-25°C). Throughout the process, standard quality control measures were carried out with caution.

Statistical analysis

The data are displayed as a mean, standard deviation, and percentages. The categorical variables were compared using the Chi-square test between cases and controls. The continuous variables were compared between the two groups using the unpaired t-test. To compare continuous variables between more than two groups, the one-way analysis of variance (ANOVA) was utilized. The sensitivity and specificity of platelet parameters for the prediction of hyperlipidemia-related disorders were determined using a receiving operating curve analysis. The p-value <0.05 was deemed significant. SPSS 21.0 was used to conduct all of the analysis (IBM Corp., Armonk, NY, USA).

## Results

The present study was intended to measure platelet parameters and function in hyperlipidemic patients and to compare the values with age and sex-matched controls having normal lipid profile results. Some 68 cases of hyperlipidemia were investigated along with 32 age and sex-matched controls.

Demographic distribution of cases and controls

The subjects were stratified into 20-29, 30-40, 41-50, and >50 years of age in both genders. The maximum number of cases (29.4%) and controls (31.25%) were in the fourth decade of life. The mean age (years) of cases and controls was 40.71 ± 15.20 and 41.4 ± 14.59 respectively. The majority of both cases (60.3%) and controls (68.8%) were males. The male to female ratio among cases and controls was 1.5 and 2.2, respectively. There was no statistically significant (p > 0.05) difference seen in age and gender between the cases and controls (Table 1).

**Table 1:** Demographic distribution of cases and controls. SD, standard deviation

Age (years)	Cases (n=68)				Controls (n=32)				p-value
				%				%	
	No. of cases	Male	Female		No. of cases	Male	Female		
<30	19	13	6	27.9	8	5	3	25.0	0.16
30-40	14	9	5	20.6	6	4	2	18.75	
41-50	20	12	8	29.4	10	8	2	31.25	
>50	15	7	8	22.1	8	5	3	25.0	
Mean ± SD	40.71 ± 15.20				41.4 ± 14.59				

In our study, out of 68 cases, 28 (41.2%) were hypertensive, 28 (41.2%) were smokers, 22 (32.4%) were alcoholics, and 15 (22.1%) were diabetics. Out of 32 controls, 12 (37.5%) were hypertensive, 12 (37.5%) were smokers, 10 (31.3%) were alcoholic, and 6 (18.8%) were diabetics. There was no statistically significant (p > 0.05) difference observed in the history between the two groups.

The mean TC (mg/dL), mean TG (mg/dL), and mean LDL (mg/dL) among cases (68) were 205.69 ± 55.51, 175.95 ± 100.89, and 116.65 ± 54.40 respectively. Controls (32) showed mean TC (mg/dL), mean TG (mg/dL), and mean LDL (mg/dL) as 124.45 ± 32.20, 87.16 ± 29.43, and 61.49 ± 25.04 respectively. All the lipid profile parameters were found to be statistically significant (p < 0.05) increased among the cases than controls.

Comparison of various platelet parameters and platelet functions between cases and controls

Among cases, the mean PC (109/L), MPV, PDW (fl), P-LCR (%), PCT (%), and platelet aggregation (%) were 193.60 ± 62.57, 10.55 ± 1.81, 14.93 ± 2.82, 30.97 ± 11.74, 0.18 ± 0.05, and 42.03 ± 25.28 respectively. In controls, mean PC (109/L), MPV, PDW (fl), P-LCR (%), PCT (%), and platelet aggregation (%) were 178.72 ± 66.74, 9.35 ± 1.85, 13.10 ± 2.60, 25.13 ± 12.23, 0.17 ± 0.05, and 31.25 ± 15.11 respectively. MPV, PDW, P-LCR, and platelet aggregation (%) with ADP showed a statistically significant increase among the cases as compared to controls (p < 0.05). There was no significant difference found for PC (p=0.28) and PCT (p=0.20) between cases and controls (Table 2).

**Table 2:** Comparison of various platelet parameters and functions between cases and controls. PC, platelet count; MPV, mean platelet volume; PDW, platelet distribution width; P-LCR, platelet large cell ratio; PCT, plateletcrit;

Platelet parameters and function	Cases (n=68) Mean ± SD	Controls (n=32) Mean ± SD	p-value
PC (10^9^/L)	193.60 ± 62.57	178.72 ± 66.74	0.28
MPV (fl)	10.55 ± 1.81	9.35 ± 1.85	0.003
PDW (fl)	14.93 ± 2.82	13.10 ± 2.60	0.003
P-LCR (%)	30.97 ± 11.74	25.13 ± 12.23	0.01
PCT (%)	0.18 ± 0.05	0.17 ± 0.05	0.20
Platelet aggregation (%)	42.03 ± 25.28	31.25 ± 15.11	0.02

Comparison of platelet parameters and functions with total cholesterol, triglycerides, and low-density lipoprotein levels

The comparison of platelet parameters and functions with TC levels displayed a statistically significant (p=0.002) increased level of PC in which high cholesterol was present (248.18 ± 36.70) than borderline cholesterol (183.91 ± 60.89). Similar observations were also found for MPV (p=0.001), PDW (p=0.004), and P-LCR (p=0.003). Mean platelet aggregation percentage was also found higher in cases with high cholesterol levels than in cases with borderline high levels but it was not statistically significant (p > 0.05). The comparison of platelet parameters and function with serum TG levels revealed a statistically significant (p=0.04) increased level of PC in whom high TG was present (206.39 ± 58.04) than borderline high TG (160.67 ± 49.48). Rest all platelet parameters were also increased in patients with high TG levels than borderline high TGs but not statistically significant (p > 0.05). All the platelet parameters were found to increase as serum LDL value increased from borderline to high to very high but were not found to be statistically significant (p > 0.05) (Table 3).

**Table 3:** Comparison of platelet parameters and functions with TC, TGs, and LPL levels. TC, total cholesterol; TGs, triglycerides; LDL, low-density lipoprotein; PC, platelet count; MPV, mean platelet volume; PDW, platelet distribution width; P-LCR, platelet large cell ratio; PCT, plateletcrit; SD, standard deviation

Lipid levels	PC (10^9^/L) Mean ± SD	MPV (fl) Mean ± SD	PDW (fl) Mean ± SD	P-LCR (%) Mean ± SD	Plateletcrit (PCT) (%) Mean± SD	Platelet aggregation (%) Mean ± SD
TC						
Borderline high (200-239 mg/dL)	183.91 ± 60.89	9.40 ± 1.01	13.19 ± 1.44	23.64 ± 6.17	0.19 ± 0.05	43.31 ± 27.35
High (≥240 mg/dL)	248.18 ± 36.70	11.25 ± 1.70	15.93 ± 2.88	35.16 ± 11.58	0.22 ± 0.04	50.27 ± 26.48
p-value	0.002	0.001	0.004	0.003	0.05	0.46
TGs						
Borderline high (150-199 mg/dL)	160.67 ± 49.48	10.30 ± 1.72	14.66 ± 2.64	29.00 ± 11.14	0.17 ± 0.04	38.11 ± 22.88
High (200-499 mg/dL)	206.39 ± 58.04	11.08 ± 1.07	15.53 ± 2.06	34.92 ± 7.63	0.19 ± 0.05	45.57 ± 27.02
p-value	0.04	0.21	0.38	0.15	0.30	0.47
LDL						
Borderline high (130-159 mg/dL)	193.50 ± 69.13	9.88 ± 1.09	13.83 ± 1.59	26.26 ± 6.69	0.19 ± 0.06	40.86 ± 25.26
High (160-189 mg/dL)	204.25 ± 37.95	10.30 ± 2.13	14.67 ± 3.43	30.22 ± 13.85	0.20 ± 0.02	52.17 ± 52.17
Very high (≥190 mg/dL)	260.67 ± 37.73	11.00 ± 1.75	15.54 ± 2.98	33.08 ± 11.67	0.24 ± 0.24	65.00 ± 26.85
p-value	0.08	0.33	0.41	0.42	0.20	0.20

## Discussion

This study was an attempt to evaluate the relation between platelet parameters and function with hyperlipidemia. Several studies have stated that PC and size might be gender and age-dependent [12-13]. Hence, we have conducted a present case-control study with both age and sex-matched controls to keep away such bias in our results. In our study, we observed a male-to-female ratio of 1.5:1 among cases which correlates with the study done by Khemka and Kulkarni and Hawaldar and Sodani [14-15].

The mean PC (109/l) in cases of hyperlipidemia was higher when compared with the mean PC of controls. However, the difference was not statistically significant (p=0.28). The Khemka et al. study [14], Ravindran and Krishnan [16], and Pathansali et al. [17] studies are also in concordance with this study. However, studies done by Jesri et al. and Prisco et al. in cases with deranged lipid profiles showed a statistically significant (p < 0.05) increase in mean PC when compared with cases of normal lipid profiles [18-19].

The MPV(fl) in cases of hyperlipidemia showed a statistically significant (p=0.003) increase when compared with the MPV of controls. The findings of our results were in concordance with the study done by Kemaka et al. [14] and Furman-Niedziejko [20]. Like the Kemaka et al. study [14], Grotto and Noronha [21], and Tseng et al. [22] studies are also found that MPV, PDW, and P-LCR were significantly higher in the study group than the control (p-value <0.05). The study done by Doğru et al. [23] and Ravindran and Krishnan [16] did not show any significant correlation between MPV and hyperlipidemia.

The PDW (fl) in cases of hyperlipidemia showed a significant (p=0.003) increase when compared with controls. The study done by Grotto and Noronha also showed a statistically significant increase in PDW in dyslipidemic patients when compared with controls [21]. The study of Ravindranand Krishnan also showed a statistically significant increase in PDW in patients with hyperlipidemia associated with coronary artery disease [16]. They actually highlighted the presence of more than one detrimental factor to have a potential effect on the platelet parameters.

In our study, the mean of P-LCR in cases of hyperlipidemia showed a statistically significant (p=0.01) increase when compared with controls. Our results were similar to the study of Khemka et al. and Grotto and Noronha [14, 21]. Larger platelets are more active, according to Grotto and Noronha study (2004), and hence contribute to vaso-occlusive events in individuals with dyslipidemia. P-LCR has been proposed as a risk factor for thromboembolic ischemic events [21].

In our study, there was a statistically significant increase in platelet aggregation observed in hyperlipidemic patients than in the control group (p < 0.05). This was comparable to the findings of Faheem et al., who found a significant positive association between platelet aggregability and cholesterol level (p=0.006) [24]. Similar findings were also noted in studies done by Kameda et al. [25], Dao et al. [26], Lakhey et al. [27], and Friend et al. [28].

We had some limitations with this study as we had a sample size of 100 patients, but the split of them into case and control groups resulted in a small sample size, which may have caused bias in the results. To confirm and validate the relevance of diverse platelet characteristics and functions in the pathophysiology of hyperlipidemia and associated illnesses, further research with bigger subgroups, additional multicentric studies, and all ethnic groups would be necessary. Our study was a prospectively recorded study, and we failed to maintain track of our patients' progress, so we have no idea what will happen to their hyperlipidemia in the future, including the incidence of any complications or related diseases.

## Conclusions

To conclude, PVI are a valuable tool for detecting bigger platelets, which are more hemostatically active and a risk factor for coronary thrombosis, and myocardial infarction in individuals with hyperlipidemia. Patients with bigger platelets who are hyperlipidemic can be easily diagnosed during normal hematological tests and may benefit from preventive medication. Platelet parameters and aggregation tests are significant, easy, effortless, and cost-effective methods for predicting oncoming acute episodes in hyperlipidemic patients that should be utilized more widely. Platelets in hyperlipidemic individuals are hyper aggregable, according to this study. To avoid vascular events, these individuals may require higher antiplatelet dosages and more rigorous hyperlipidemia therapy.
